# TriLock-FISH enables *in situ* discrimination of influenza cRNA and mRNA for single-cell analysis of viral replication and transcription

**DOI:** 10.1128/mbio.01572-26

**Published:** 2026-10-22

**Authors:** Lingkai Zhang, Wei Chen, Xinyu Gao, Huaiyuan Cai, Xu Zhou, Rizwan Ullah, Xinyi Pei, Jinxia Dai, Gang Cao, Guiqing Peng, Hongbo Zhou, Wen Su

**Affiliations:** 1 National Key Laboratory of Agricultural Microbiology, College of Veterinary Medicine, Huazhong Agricultural University 47895, Wuhan, Hubei, China; 2 Hubei Hongshan Laboratory, Wuhan, Hubei, China; 3 Frontiers Science Center for Animal Breeding and Sustainable Production, Wuhan, Hubei, China; 4 The Cooperative Innovation Center for Sustainable Pig Production, Huazhong Agricultural University 47895, Wuhan, Hubei, China; 5 Shennongjia Science and Technology Innovation Center, Shennongjia Multidisciplinary Observation and Research Station of the Ministry of Agriculture and Rural Affairs, Huazhong Agricultural University 47895, Wuhan, Hubei, China; University of Wisconsin-Madison, Madison, Wisconsin, USA

**Keywords:** influenza A virus, fluorescence *in situ* hybridization, single-cell RNA imaging, time-resolved infection dynamics, viral RNA replication

## Abstract

Influenza A virus produces three RNA species—viral RNA (vRNA), complementary RNA (cRNA), and messenger RNA (mRNA)—whose synthesis and trafficking are spatially compartmentalized. However, the high sequence similarity between cRNA and mRNA hinders their distinction *in situ*, limiting understanding of the spatial organization of the viral replication-transcription dynamics. Here, triple-lock fluorescence *in situ* hybridization (TriLock-FISH) was developed to distinguish vRNA, cRNA, and mRNA *in situ* with high specificity. TriLock-FISH enabled a genome-wide, time-resolved single-cell atlas of RNA abundance and localization, revealing marked cell-to-cell variation in RNA abundance but relatively conserved spatial organization. cRNA was found to be predominantly nuclear, with a small but reproducible cytoplasmic fraction. Application to infected mouse lung tissue demonstrated simultaneous spatial detection of all viral RNA species. This approach provides a highly specific framework for single-cell analysis of RNA virus replication and transcription.

**IMPORTANCE:**

Influenza virus relies on three closely related RNA molecules for replication and gene expression, yet distinguishing them inside infected cells has remained technically challenging. This limitation has hindered understanding of viral replication in space and time. We developed triple-lock fluorescence *in situ* hybridization, which enables simultaneous visualization of all three viral RNA species across all eight influenza genome segments in infected cells and tissues. Single-cell analysis revealed striking heterogeneity in viral RNA abundance across infected cells, whereas intracellular RNA localization patterns remained relatively conserved. Complementary RNA, a replication intermediate long thought to be strictly nuclear, was also detected at low levels in the cytoplasm. These findings provide new insight into influenza virus replication and establish a broadly applicable tool for studying RNA virus biology.

## INTRODUCTION

Influenza A virus (IAV), a member of the Orthomyxoviridae family, remains a persistent global health threat (1). Its replication involves three RNA species synthesized in the host nucleus: genomic viral RNA (vRNA), complementary RNA (cRNA), and messenger RNA (mRNA) (2). vRNA is the packaged genome and template for RNA synthesis; cRNA serves as a replicative intermediate for vRNA amplification; and mRNA directs viral protein translation via a cap-snatching mechanism (3). The spatial coordination of RNA synthesis, nuclear retention, export, and cytoplasmic transport is central to efficient viral replication and genome packaging (4).

Considerable effort has been devoted to quantifying viral RNA synthesis. Early techniques such as northern blotting were laborious and insensitive (5–7). Strand-specific RT-qPCR improved quantification and revealed population-level kinetics (8, 9). Strand-specific total RNA sequencing (e.g., InVERT) enabled genome-wide, parallel analysis of all three RNA types, providing insights into segment-specific regulation (10). However, these bulk methods average signals across cell populations, masking single-cell heterogeneity—a hallmark of infection (11–15). While single-cell RNA sequencing (scRNA-seq) resolves cellular heterogeneity in host response and viral burden, it cannot natively distinguish the three similar viral RNA species and lacks spatial information (12, 15).

Spatial context is crucial, as the IAV life cycle is compartmentalized. RNA fluorescence *in situ* hybridization (FISH), particularly single-molecule FISH (smFISH), has visualized nuclear export (16–18), cytoplasmic assembly via Rab11 (19, 20), and trafficking of vRNA segments (21), as well as mRNA export mechanisms (22, 23). A persistent gap has been the reliable *in situ* detection of cRNA. Its near-complete sequence identity with mRNA—differing only at short termini—makes it indistinguishable using conventional FISH probes. Consequently, studies of viral mRNA may have inadvertently detected cRNA, and direct cRNA visualization has required indirect strategies like transcriptional inhibition (24). As a result, the spatial dynamics of cRNA and the complete single-cell replication–transcription program remain incompletely characterized.

Here, we developed triple-lock fluorescence *in situ* hybridization (TriLock-FISH), a triplex probe–based strategy that decouples RNA class recognition from segment-specific identification and integrates them through a dual-ligation mechanism. Only when three probes correctly assemble on the target RNA molecule can rolling circle amplification (RCA) be initiated, thereby enabling precise discrimination of vRNA, cRNA, and mRNA *in situ*, including single-nucleotide variants. After *in vitro* validation, we applied TriLock-FISH to infected cells to construct a quantitative, genome-wide single-cell spatial atlas of viral RNAs across all eight segments. Viral abundance varied markedly between cells, whereas subcellular localization patterns remained relatively conserved. Time-resolved analysis further defined the coordinated dynamics of RNA synthesis, nuclear retention, and cytoplasmic redistribution during early infection. Finally, application to infected lung tissue demonstrated simultaneous spatial resolution of all three RNA species across all eight genomic segments *in vivo*. Together, these findings reveal a spatially organized replication–transcription program and establish TriLock-FISH as a framework for dissecting RNA virus replication within complex biological contexts.

## RESULTS

### Development and validation of the TriLock-FISH method

**Fig 1:**
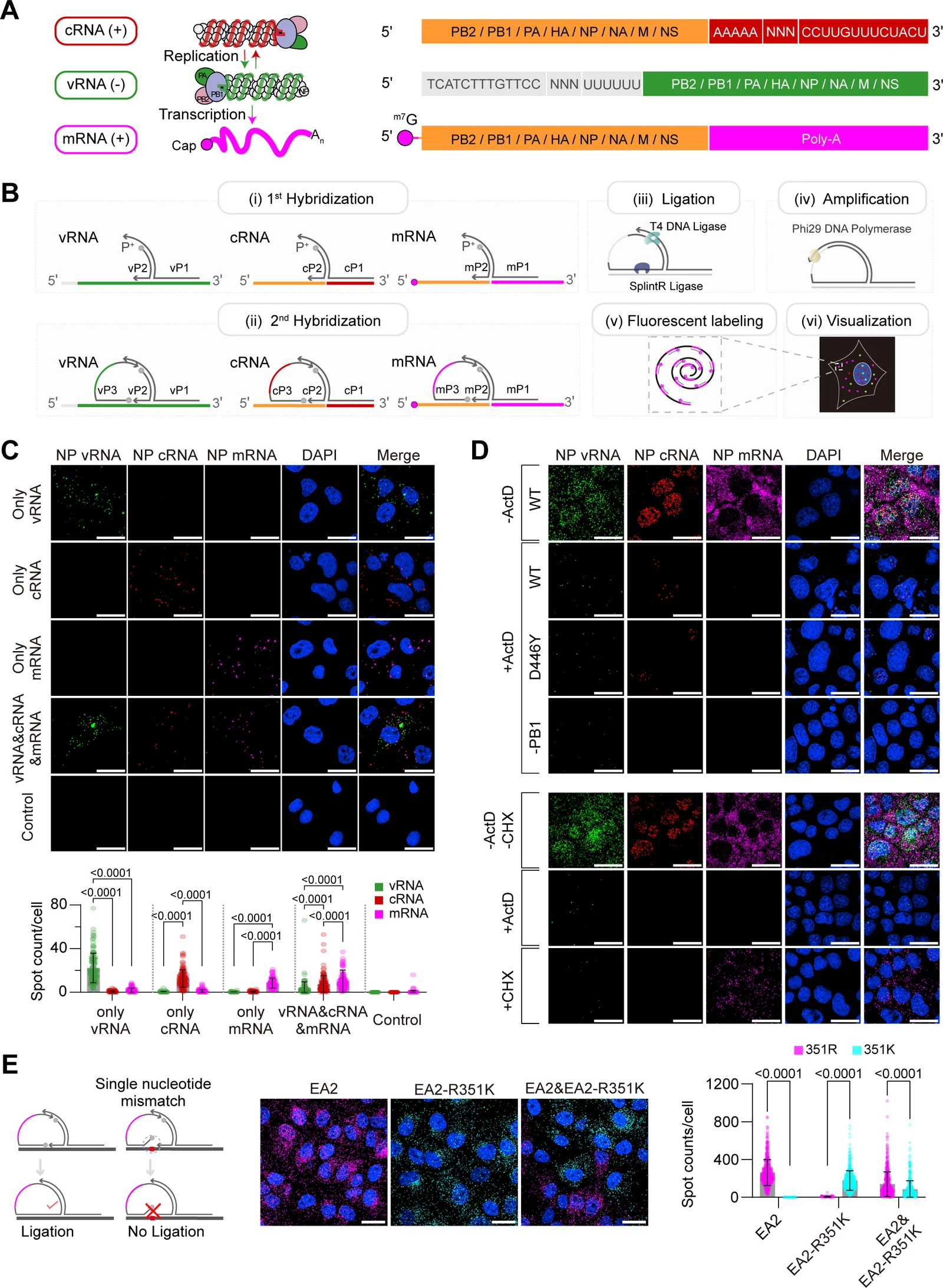
Principle and validation of TriLock-FISH for *in situ* detection of influenza A virus vRNA, cRNA, and mRNA. (**A**) Schematic of the three IAV RNA species. vRNA (−) templates cRNA and mRNA (+), which share segment-specific internal sequences but differ at the 3′ terminal. (**B**) TriLock-FISH workflow. A pre-annealed probe P1–P2 duplex first hybridizes to the vRNA internal sequence, conserved cRNA 3′ end, or the mRNA poly(A) junction region. P3 then binds an adjacent target sequence to complete a ligation-competent complex. Correctly assembled probes undergo ligation, rolling circle amplification, and fluorescent detection. (**C**) Specificity validation using defined RNAs. T7-transcribed A/Puerto Rico/8/1934 (PR8) NP vRNA, cRNA, and/or mRNA (50 ng per condition) were transfected into MDCK cells and analyzed at 4 h post-transfection (hpi). Representative images and per-cell spot counts are shown. vRNA, Alexa 488 (green); cRNA, Alexa 546 (red); mRNA, Alexa 647 (magenta); and nuclei, DAPI (blue). Error bars represent the mean ± SD. Data are representative of at least two independent experiments. *P* values were calculated using the Friedman test followed by Dunn’s *post hoc* test. Scale bars, 25 μm. (**D**) Validation of drug-treated replication and cRNP stabilization assays. MDCK cells were either pre-transfected with PR8 RNP plasmids (upper four rows) or left non-transfected (lower three rows), treated as indicated, and infected with PR8 (MOI = 5) for 5 h before TriLock-FISH. Transfected groups included WT control (PB2, PB1, PA, and NP; −ActD), replication assay (WT; +ActD), cRNP stabilization assay (D446Y: PB2, PB1 D446Y, PA, and NP), and negative control (−PB1: PB2, PA, and NP). Non-transfected groups included untreated (−ActD/−CHX), +ActD, and +CHX conditions. Representative images from at least two independent experiments are shown. Scale bars, 25 μm. (**E**) Single-nucleotide specificity validation. MDCK cells were infected with EA2, EA2 NP-R351K, or a mixture of both (MOI = 0.5) for 9 h. Quantification (right) shows allele-specific detection by TriLock-FISH. Error bars represent the mean ± SD. Data are representative of at least two independent experiments. *P* values were calculated using the Mann-Whitney test.

A key challenge in studying the spatial organization of the influenza A virus replication cycle is distinguishing its three RNA species (vRNA, cRNA, and mRNA) *in situ*. While vRNA can be distinguished by strand orientation, cRNA and mRNA share near-identical internal sequences but differ at their short class-specific termini, making them indistinguishable by conventional padlock probes (Fig. 1A; Fig. S1A and B). The conserved cRNA 3′ terminus does not encode segment identity, whereas the poly(A) tail of mRNA is indistinguishable from host transcripts. Thus, accurate *in situ* detection requires simultaneous resolution of RNA class (vRNA, cRNA, or mRNA) and segment origin.

To meet this dual requirement, we developed the TriLock-FISH, a triplex probe strategy that converts RNA-type recognition and segment identification into a ligation-dependent amplification event (Fig. 1B). Three probes (P1–P3) co-assemble on a single RNA molecule to form a circularizable complex. P1 binds an RNA class–defining feature, whereas P2 and P3 hybridize to adjacent segment-specific internal sequences (Fig. S2). Only correctly assembled P1/P2/P3 triplets undergo dual ligation and rolling circle amplification (RCA), ensuring that signal is generated only when both RNA class and segment identity are correctly matched (Fig. S1C). This design prevents signal generation from partially matched or cross-reactive hybridization events (Fig. S3). TriLock-FISH builds upon principles from previously reported high-specificity *in situ* detection approaches (18, 25–29).

Probe specificity was first validated using defined RNA substrates. MDCK cells transfected with individually *in vitro*-transcribed NP-segment vRNA, cRNA, or mRNA showed highly specific detection with minimal cross-reactivity (Fig. 1C). To further validate specificity during infection, drug perturbation was combined with replication and cRNP stabilization assays adapted from a previous study (24). In non-transfected cells, cycloheximide (CHX; blocking protein synthesis) treatment permitted robust detection of primary mRNA but strongly suppressed cRNA accumulation, whereas actinomycin D (ActD; inhibiting transcription) abolished mRNA and cRNA signals, leaving only sparse vRNA puncta (Fig. 1D). Under ActD-treated replication assay conditions (wild-type [WT] polymerase), both vRNA and cRNA accumulated without detectable mRNA. Under cRNP stabilization conditions, the catalytically dead mutant (PB1 D446Y), which stabilizes cRNPs but prevents further replication, retained clear cRNA signals, with only sparse vRNA puncta and no mRNA. These results confirm that TriLock-FISH reliably distinguishes mRNA from cRNA during infection.

TriLock-FISH also distinguished infection by a wild-type Eurasian avian-like H1N1 swine influenza virus (EA2) from its isogenic mutant (EA2 R351K) differing by a single nucleotide within the probe target site, demonstrating single-nucleotide specificity *in situ* (Fig. 1E). Systematic negative controls confirmed that omission or mismatch of probe components failed to generate amplification signals (Fig. S4).

These results support that TriLock-FISH achieves strand- and segment-specific *in situ* discrimination of vRNA, cRNA, and mRNA through coordinated probe assembly and dual-ligation control.

### Optimization and validation of TriLock-FISH in cellular models

**Fig 2:**
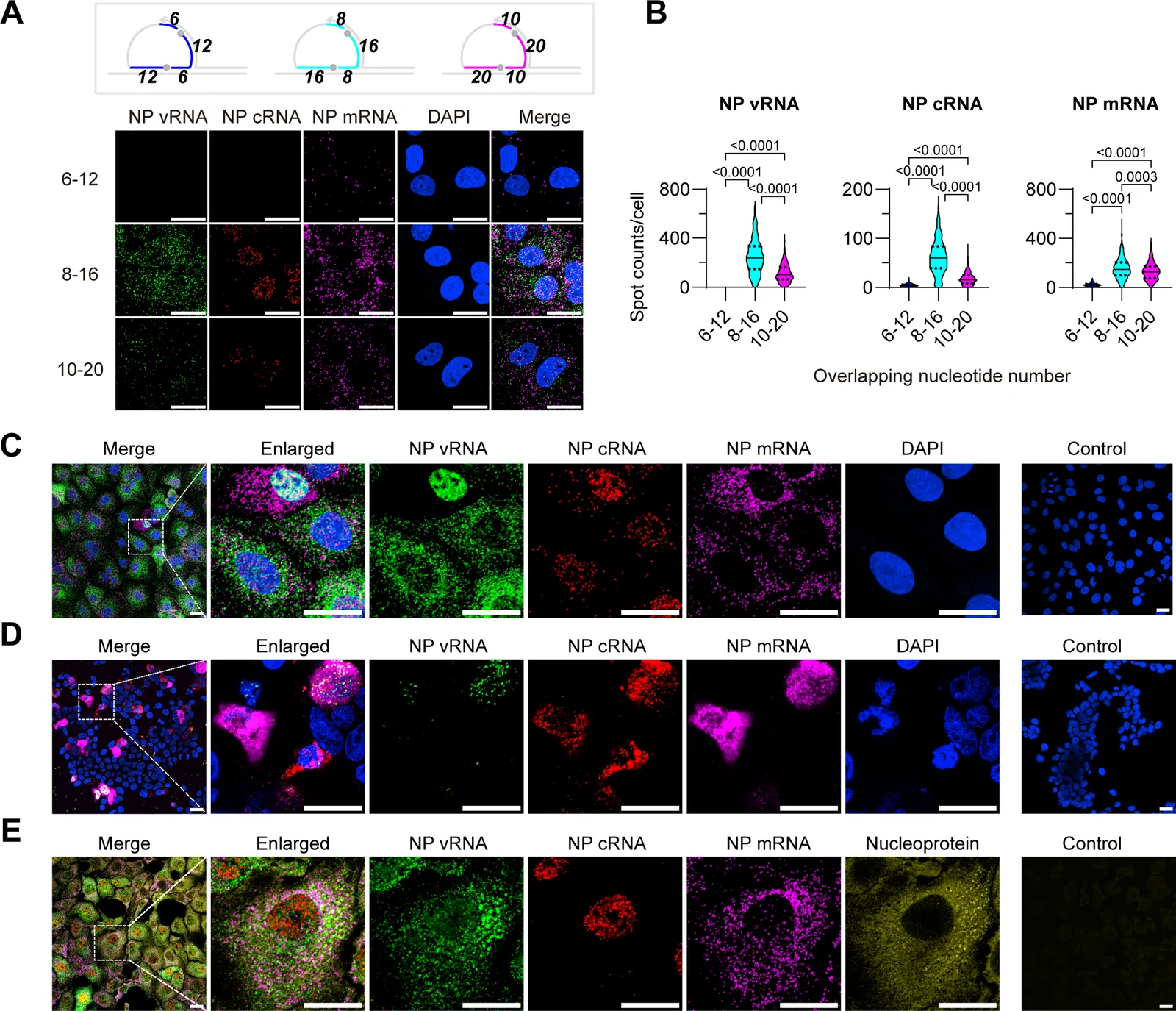
Optimization and validation of TriLock-FISH in cellular systems. (**A**) Comparison of probe architectures for hybridization efficiency by detecting IAV NP RNA in PR8-infected MDCK cells at 8 hpi (MOI = 1). (**B**) Quantification of mean spot counts per cell from panel A. The *P* values were calculated by the Kruskal–Wallis test, followed by Dunn’s *post hoc* test. Data are representative of two independent experiments. (**C**) Detection of NP vRNA, cRNA, and mRNA in PR8-infected MDCK cells at 8 hpi (MOI = 1). (**D**) HEK293T cells were transfected with plasmids encoding PB1, PB2, PA, and NP of H1N1 (PR8). NP vRNA, cRNA, and mRNA produced by the reconstituted replication machinery were detected at 24 h post-transfection. (**E**) Simultaneous detection of NP RNA and NP protein in PR8-infected MDCK cells at 8 hpi (MOI = 1). Nucleoprotein, DyLight 405 (yellow); vRNA, Alexa 488 (green); cRNA, Alexa 546 (red); and mRNA, Alexa 647 (magenta). Scale bars, 25 μm.

Following validation, we optimized probe architecture to enhance detection efficiency under infection conditions. Comparative analysis identified an “8-16” overlap configuration as providing the strongest signal while maintaining specificity (Fig. 2A and B). Hybridization parameters were further refined to improve robustness (Fig. S5), establishing a standardized workflow for subsequent applications. To benchmark the quantitative performance of TriLock-FISH, we compared vRNA detection with two established padlock probe-based methods—cDNA padlock probe hybridization and direct RNA padlock probe hybridization—and found that TriLock-FISH yielded higher vRNA spot counts per cell (Fig. S6).

We then evaluated TriLock-FISH across multiple cellular contexts. In PR8-infected MDCK cells, vRNA, cRNA, and mRNA were simultaneously detected with distinct and expected subcellular distributions (Fig. 2C), confirming that the triplex assembly logic functions during authentic infection. In a plasmid-driven mini-replicon system in 293T cells, the method detected newly synthesized vRNA and cRNA generated by reconstituted viral polymerase complexes (Fig. 2D), demonstrating compatibility with replication assays independent of full viral infection.

TriLock-FISH was further combined with immunofluorescence to enable concurrent visualization of viral RNA and protein within the same cell (Fig. 2E; Fig. S7). Additionally, endogenous host GAPDH mRNA was successfully detected (Fig. S8), indicating that the strategy is applicable beyond viral RNAs. These results demonstrate that TriLock-FISH operates robustly across diverse cellular models and experimental settings.

### Genome-wide single-cell analysis reveals conserved spatial organization of influenza viral RNAs

**Fig 3:**
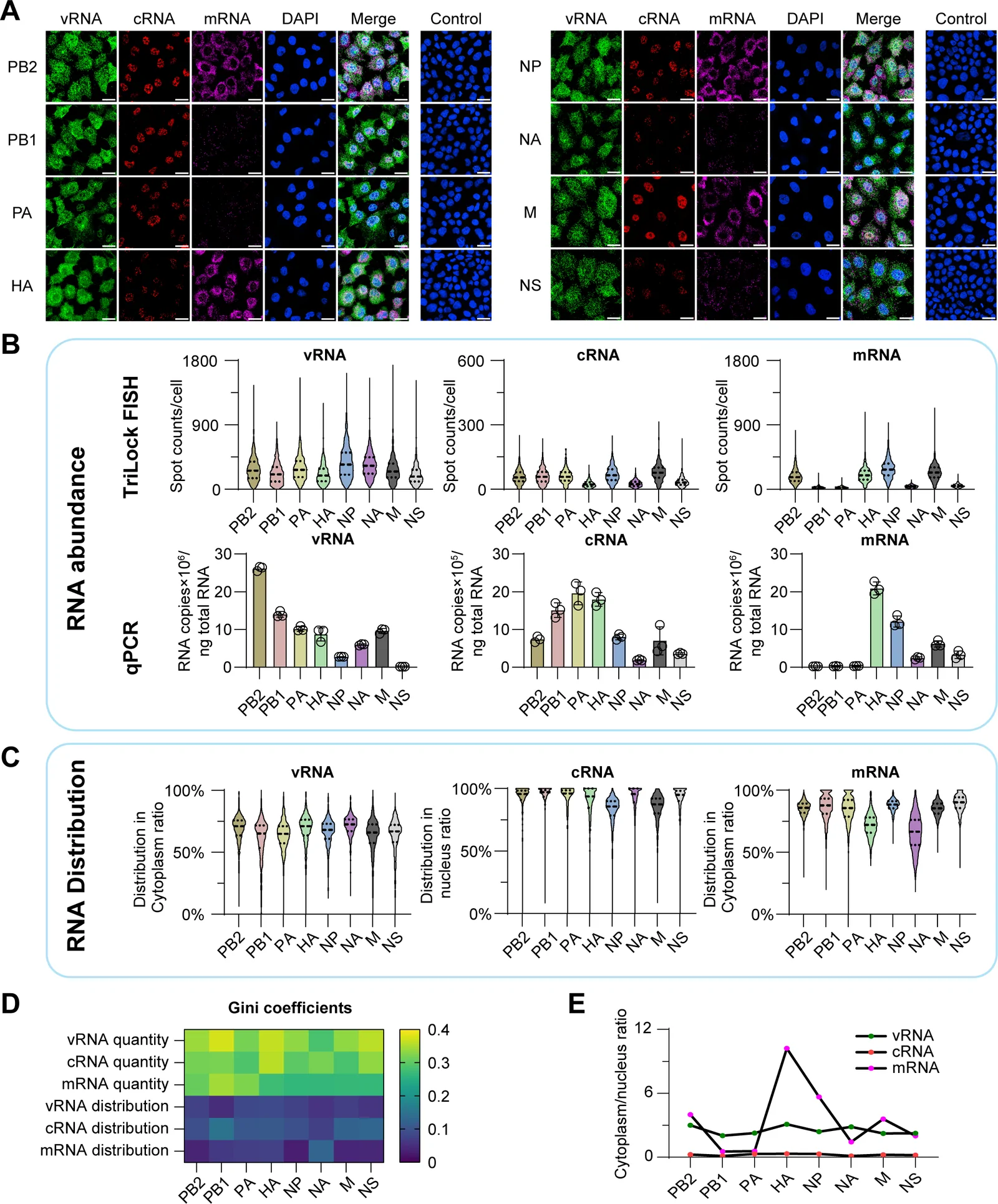
Genome-wide quantitative and spatial profiling of influenza viral RNAs at single-cell resolution. (**A**) Visualization of the IAV genome and transcriptome. MDCK cells infected with PR8 (MOI = 0.5) were analyzed by TriLock-FISH at 6 hpi using probe sets targeting all eight viral segments. Scale bars, 25 μm. Data are representative of three independent experiments. (**B**) Quantification of viral RNA abundance across all eight segments by TriLock-FISH (top) and strand-specific RT-qPCR (bottom). For TriLock-FISH, per-cell spot counts of vRNA, cRNA, and mRNA are shown as violin plots (*n* ≥ 700 cells per segment from three independent experiments). For RT-qPCR, average copy numbers of vRNA, cRNA, and mRNA per ng total RNA were determined from three parallel samples prepared under identical infection conditions. Error bars represent mean ± SD from three independent experiments. (**C**) Segment-resolved subcellular distribution of each RNA species, shown as cytoplasmic fractions for vRNA and mRNA and nuclear fractions for cRNA. (**D**) Gini coefficient heatmap of vRNA, cRNA, and mRNA across all eight segments, quantifying cell-to-cell inequality in RNA abundance and subcellular distribution as measured by TriLock-FISH. (**E**) Segment-resolved mean cytoplasm-to-nucleus ratios of vRNA, cRNA, and mRNA.

To examine the spatial organization of viral RNA synthesis at genome scale, TriLock-FISH was applied to simultaneously detect vRNA, cRNA, and mRNA for all eight segments in PR8-infected MDCK cells at 6 hpi (Fig. 3A). For M and NS, probes targeted transcripts sharing the conserved 3′ terminal region. Segment-specific probe sets enabled visualization of the complete viral RNA complement within individual cells, allowing simultaneous analysis of RNA abundance and subcellular localization.

Single-cell analysis revealed distinct quantitative and spatial patterns among the three RNA species (Fig. 3B and C). Across most segments, vRNA was generally the most abundant species, whereas cRNA exhibited the lowest abundance. mRNA displayed intermediate abundance and was predominantly cytoplasmic. Despite large variability in RNA abundance between infected cells, RNA localization patterns were consistent across segments: vRNA and mRNA were mainly cytoplasmic, whereas cRNA was enriched in the nucleus. Notably, a small but reproducible cytoplasmic fraction of cRNA was also detected, indicating that cRNA may not be strictly confined to the nucleus. Parallel strand-specific RT-qPCR, as previously described (9), showed broadly consistent RNA-class trends, although differences were observed for a few segments (Fig. 3B).

To quantitatively compare heterogeneity in RNA abundance and spatial distribution across viral genome segments, Gini coefficients were calculated across infected cells (Fig. 3D). Gini coefficients, previously used to measure the inequality of viral transcription and replication across infected cells, range from 0 (complete equality) to 1 (maximal inequality) and provide a scale-independent measure of inequality across variables with different numerical ranges (30). RNA abundance displayed relatively high Gini values, indicating substantial cell-to-cell variability, whereas spatial distribution patterns showed lower Gini coefficients, suggesting conserved localization behavior despite quantitative heterogeneity. Consistent with these observations, segment-averaged cytoplasm-to-nucleus ratios revealed distinct spatial biases among RNA classes (Fig. 3E): vRNA showed relatively uniform ratios across segments, cRNA remained strongly nuclearly enriched, whereas mRNA displayed greater variability.

### Time-resolved single-cell analysis uncovers coordinated dynamics of viral RNA synthesis and trafficking

**Fig 4:**
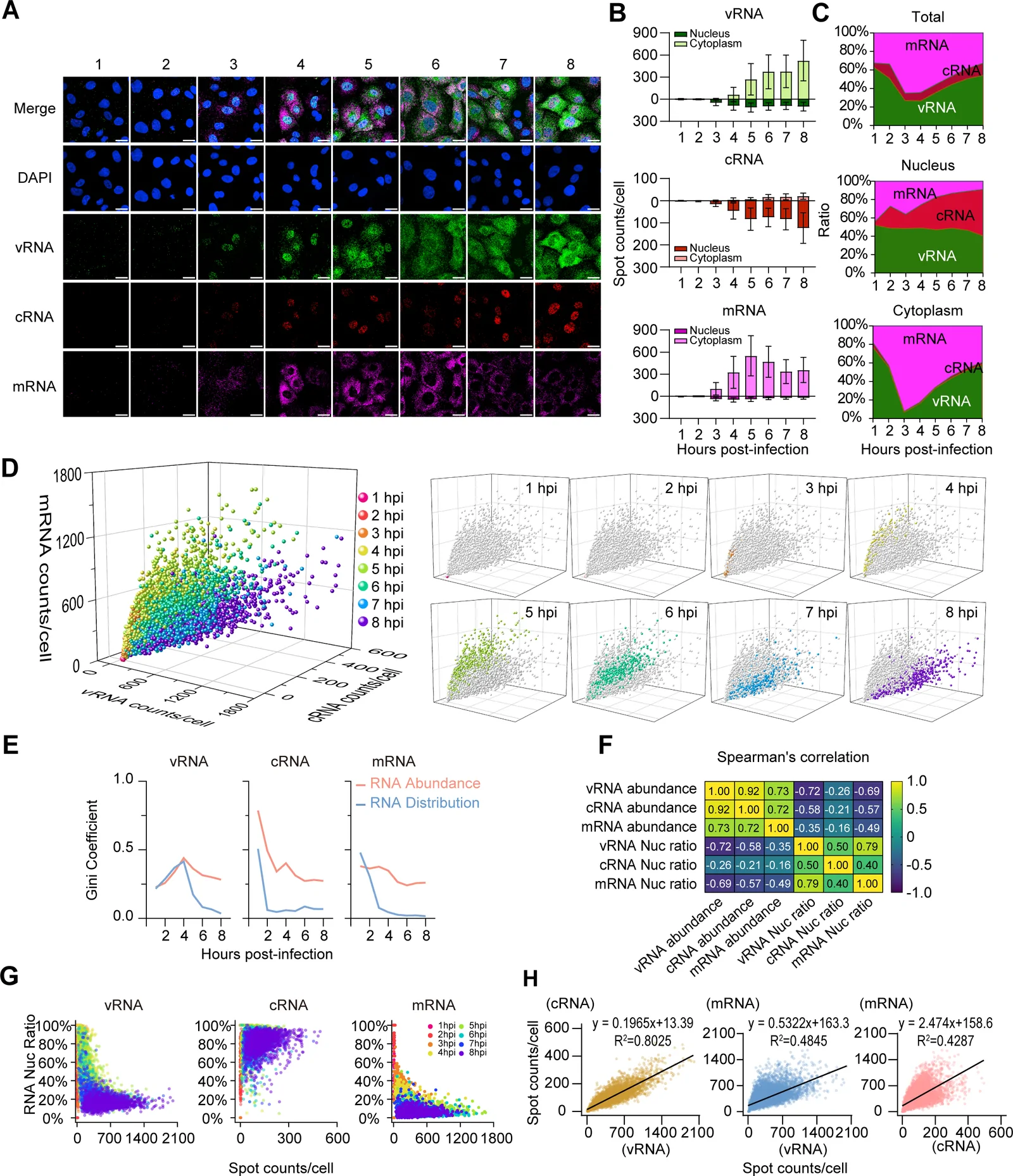
High-resolution spatiotemporal dynamics of NP-segment vRNA, cRNA, and mRNA at single-cell resolution. (**A**) Representative TriLock-FISH images from 1 to 8 hpi. MDCK cells infected with PR8 (MOI = 1) were fixed at the indicated time points. Data are representative of two independent experiments; 6,334 cells were analyzed in total. Scale bars, 25 μm. (**B**) Per-cell spot counts of NP vRNA, cRNA, and mRNA in nuclear and cytoplasmic compartments across time. Error bars represent the mean ± SD. (**C**) Stacked area plots showing the mean relative contributions of vRNA, cRNA, and mRNA to total viral RNA load, and within nuclear and cytoplasmic compartments, over time. (**D**) Single-cell embedding in a 3D scatter plot defined by vRNA, cRNA, and mRNA abundances, visualizing the dynamic shifts shown in panels B and C. Left, all cells colored by hours post-infection. Right, the same embedding shown as time-sliced views. (**E**) Time-resolved Gini coefficients quantifying cell-to-cell heterogeneity in RNA abundance and spatial distribution for vRNA, cRNA, and mRNA. Spatial distribution was calculated using the cytoplasmic fraction for vRNA and mRNA and the nuclear fraction for cRNA. (**F**) Spearman’s correlation matrix relating RNA abundance and nuclear fraction across single cells. (**G**) Single-cell distributions of viral RNA abundance and subcellular localization for vRNA, cRNA, and mRNA. Each point represents an individual cell and is color-coded by time post-infection (hpi). (**H**) Pairwise abundance relationships among RNA species at the single-cell level. Each dot represents one cell, and lines indicate linear fits.

To characterize how viral RNA synthesis and localization evolve during infection, we performed a time-resolved analysis of NP-segment vRNA, cRNA, and mRNA from 1 to 8 hpi using TriLock-FISH (Fig. 4A). The three RNA species showed distinct temporal accumulation patterns (Fig. 4B; Fig. S9A through E). vRNA increased progressively and shifted from early nuclear enrichment to cytoplasmic accumulation after ~3 hpi. cRNA accumulated gradually and remained strongly nuclear-enriched (>80%) throughout infection. In contrast, mRNA rose rapidly during early infection, peaked in the cytoplasm around 5 hpi, and then declined. These dynamics produced clear temporal shifts in RNA composition (Fig. 4C). At the whole-cell level, mRNA dominated early infection, whereas vRNA increased at later stages. Within the nucleus, mRNA progressively decreased over time as cRNA became predominant, while in the cytoplasm, mRNA dominance was gradually replaced by vRNA accumulation. A small but consistent fraction of cytoplasmic cRNA (ccRNA) was also detected and increased modestly during infection (Fig. S9E). To examine the relationship between ccRNA and viral RNA abundance, we performed single-cell linear regression analyses. ccRNA abundance showed no strong linear association with vRNA, cRNA, or mRNA abundance, with all *R*^2^ values below 0.5 (Fig. S9F).

Despite this overall temporal progression, substantial cell-to-cell heterogeneity was observed at the single-cell level. When cells were plotted in a three-dimensional (3D) coordinate space defined by vRNA, cRNA, and mRNA abundance, they distributed along a continuum of infection states (Fig. 4D). Early time points (1, 2, and 3 hpi) were characterized by low RNA levels, whereas cells from later stages progressively shifted toward regions of higher RNA abundance, forming a temporal trajectory of infection states. To examine whether similar patterns existed in spatial organization, we performed a complementary three-dimensional embedding based on RNA localization metrics, which revealed continuous changes in subcellular RNA distribution across time (Fig. S10).

To compare heterogeneity in RNA abundance and spatial distribution over time, we calculated time-resolved Gini coefficients for the NP segment across infection stages (Fig. 4E). Across all three RNA species, Gini values for RNA abundance remained higher than those for spatial distribution, indicating strong cell-to-cell heterogeneity in RNA quantity but comparatively uniform subcellular localization patterns. Consistent with the genome-wide analysis in Fig. 3, the spatial organization of vRNA, cRNA, and mRNA remained relatively stable across cells at each infection stage, suggesting a temporally conserved viral RNA distribution.

We next examined the relationship between viral RNA abundance and subcellular localization by analyzing pairwise correlations across single cells. Spearman’s correlation analysis showed strong positive correlations among the abundances of vRNA, cRNA, and mRNA (vRNA–cRNA *r* = 0.92; vRNA–mRNA *r* = 0.73; and cRNA–mRNA *r* = 0.72) (Fig. 4F). The nuclear fraction of each RNA was generally negatively correlated with its abundance (Fig. 4F). Consistent with this pattern, single-cell distributions showed that vRNA and mRNA tended to have lower nuclear fractions at higher abundance, while cRNA remained largely nuclear regardless of abundance. Data points were color-coded by time post-infection to visualize these patterns over the course of infection (Fig. 4G; Fig. S11).

Finally, pairwise relationships among RNA abundances were examined (Fig. 4H; Fig. S12). Across these cells, vRNA and cRNA showed a strong linear correlation (*R*^2^ ≈ 0.80), consistent with their template–product relationship during genome replication, whereas correlations involving mRNA were weaker (*R*^2^ ≈ 0.42–0.48). Time-resolved analysis showed low correlations among all three RNA species at 1–2 hpi (*R*^2^ < 0.2) (Fig. S12A through C). From 3 hpi onward, vRNA and cRNA remained strongly correlated (*R*^2^ ≈ 0.61–0.80), while correlations involving mRNA progressively declined. Consistently, mRNA-related slopes peaked early and decreased over time, whereas the vRNA–cRNA slope remained relatively stable, indicating an early transcription-dominant phase followed by sustained genome replication (Fig. S12D).

Collectively, these results define a coordinated spatiotemporal program of influenza viral RNA metabolism: rapid mRNA export supports early protein synthesis, nuclear-retained cRNA templates genome replication, and vRNA shifts from the nucleus to the cytoplasm for assembly. Despite large variability in RNA abundance among single cells, the spatial organization of viral RNAs remains relatively consistent.

### Genome-wide *in situ* detection of influenza viral RNAs in infected lung tissue

**Fig 5:**
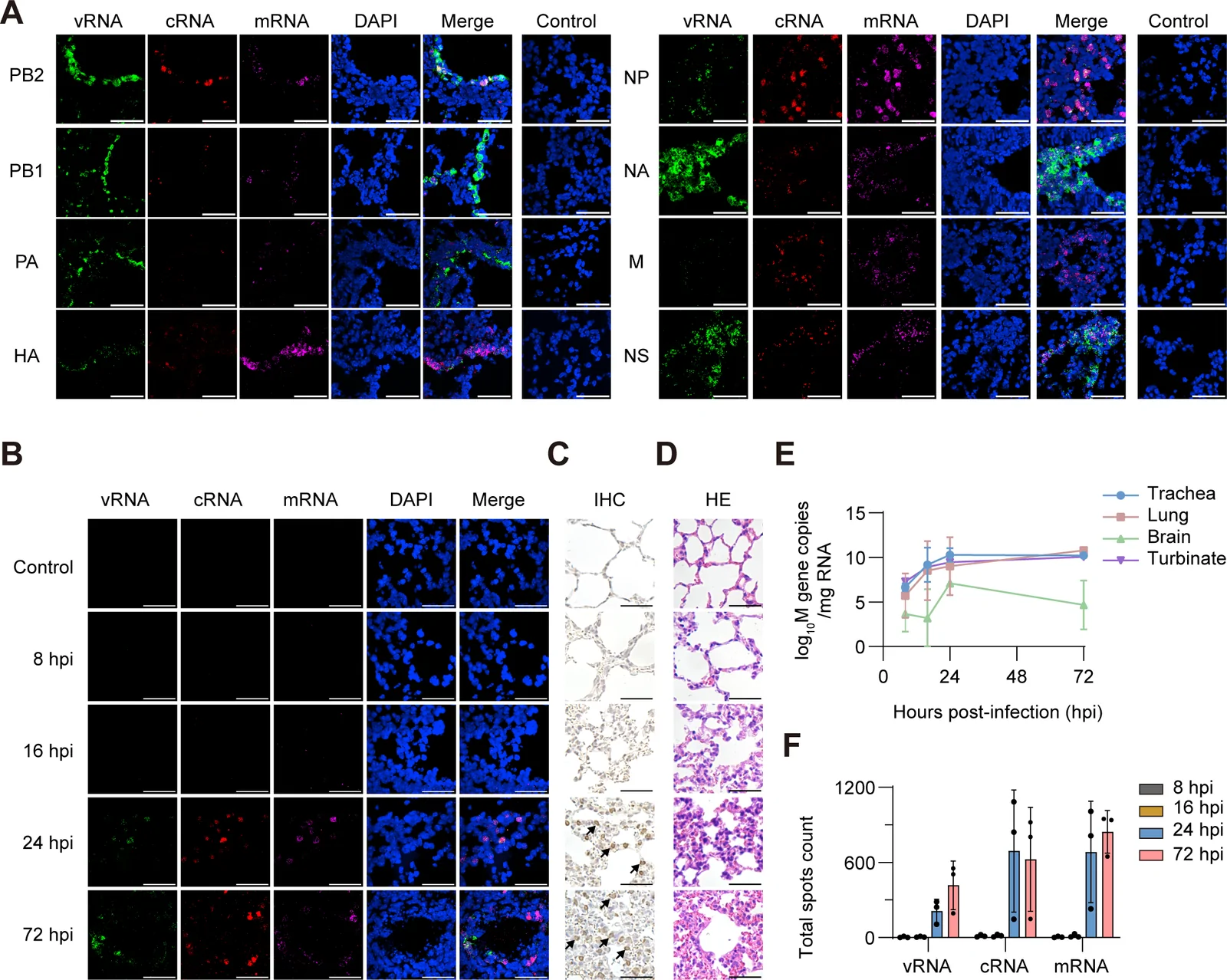
*In situ* visualization and validation of viral genome and transcriptome in IAV-infected mouse lung tissue. Female BALB/c mice were intranasally inoculated with 10^5^ PFU PR8, and tissues were collected at the indicated time points (*n* = 3 mice per time point). (**A**) TriLock-FISH detection of vRNA, cRNA, and mRNA for all eight segments in lung sections at 72 hpi; nuclei are stained with DAPI. “Control” indicates the negative control. (**B**) Longitudinal analysis of the NP segment. Time-course TriLock-FISH detection of NP-segment vRNA, cRNA, and mRNA in lung sections collected at 8, 16, 24, and 72 hpi (and uninfected control). (**C**) Immunohistochemistry (IHC) for viral NP in lung sections at the indicated time points; black arrows mark NP-positive staining. (**D**) H&E staining of lung sections at the indicated time points. (**E**) Viral loads measured by TaqMan RT-qPCR for the total M-segment RNA in trachea, lung, nasal turbinate, and brain, expressed as log_10_ copies per mg of total RNA. Error bars represent the mean ± SD. (**F**) Semi-quantification of TriLock-FISH puncta in lung sections over time. For each time point, *n* = 3 mice; for each mouse, three random 60× fields were imaged, and RNA puncta were summed per field for vRNA, cRNA, and mRNA. Error bars represent the mean ± SD. Scale bars, 50 μm.

We next applied TriLock-FISH to lung sections from PR8-infected mice to determine whether viral RNA species can be resolved at genome scale *in vivo*. At 72 hpi, the method simultaneously detected vRNA, cRNA, and mRNA for all eight viral genomic segments in lung tissue (Fig. 5A), demonstrating genome-wide *in situ* detection of the viral RNA complement in infected tissue.

A longitudinal analysis of the NP segment showed that specific signals for all three RNA species were detectable at 24 hpi and increased markedly by 72 hpi (Fig. 5B). This temporal profile coincided with the onset of viral nucleoprotein detection by immunohistochemistry (IHC) (Fig. 5C) and with the progression of histopathological injury observed in H&E-stained sections (Fig. 5D). Quantitative RT-PCR independently showed a sharp increase in total M-segment RNA levels in lung tissue beginning at 24 hpi (Fig. 5E), consistent with the timing of RNA detection by TriLock-FISH. Semi-quantitative analysis of TriLock-FISH signal puncta in tissue confirmed a significant increase from 24 to 72 hpi (Fig. 5F).

These results demonstrate that TriLock-FISH enables direct *in situ* detection of influenza viral genome replication and transcription in infected tissues, extending spatial analysis of viral RNA dynamics from cultured cells to the *in vivo* context.

## DISCUSSION

Understanding the spatiotemporal dynamics of influenza A virus RNAs has been limited by methodological constraints. Bulk approaches provide population averages but lack spatial resolution, while single-cell methods cannot distinguish vRNA, cRNA, and mRNA (8–15). FISH studies have revealed vRNP trafficking and viral mRNA export, yet the near-identical sequences of cRNA and mRNA prevent reliable discrimination using conventional probes (16–23). To overcome this limitation, TriLock-FISH was developed to enable highly specific *in situ* discrimination of vRNA, cRNA, and mRNA without requiring reverse transcription.

A key finding of this study is that influenza infection exhibits pronounced cell-to-cell heterogeneity in viral RNA abundance, accompanied by relatively conserved subcellular distribution patterns. This heterogeneity became more evident at later stages of infection (6–8 hpi). This suggests that although replication efficiency varies substantially between infected cells, intracellular RNA trafficking follows a tightly regulated program linked to progression through the viral life cycle. One potential regulator of this coordination is the NS segment, whose products NS1 and NS2 modulate the transcription–replication balance, while NS2 mediates vRNP nuclear export (31, 32). Such regulation may help synchronize infection progression across cells despite large differences in viral RNA output.

Direct visualization of cRNA also provides new insight into influenza replication biology. Although cRNA has traditionally been regarded as a strictly nuclear replication intermediate, we consistently detected a small but reproducible cytoplasmic fraction. Previous studies have suggested possible cytoplasmic cRNA, and our findings provide direct spatial evidence supporting this possibility (33, 34). Single-cell linear regression analyses revealed no strong linear associations between cytoplasmic cRNA (ccRNA) abundance and the abundance of vRNA, cRNA, or mRNA, although indirect or stage-specific relationships cannot be excluded. Whether ccRNA reflects transient export, mislocalized cRNPs, or intermediates involved in host sensing remains unclear. Further studies investigating the cellular factors and organelles associated with ccRNA, including systematic colocalization analyses and perturbation of nuclear export using leptomycin B, may help elucidate its subcellular distribution and potential biological significance (16–18, 33, 35).

Compared with previously reported methods, TriLock-FISH demonstrated robust detection of influenza viral RNA, supporting its utility for quantitative single-cell analysis. However, like other FISH-based approaches, it does not provide absolute RNA quantification. Differences between TriLock-FISH and RT-qPCR measurements may reflect both their distinct measurement scales—single-cell vs bulk—and method-specific biases, including RNA secondary structure, probe accessibility, and signal amplification efficiency. Consequently, the two assays should be considered complementary rather than directly interchangeable. For influenza RNA, NP binding may further affect probe accessibility. Although RNAfold predictions informed vRNA probe design, further optimization remains possible (36). In contrast, the target regions for cRNA- and mRNA-specific probes are restricted to specific terminal sequences, limiting the scope for probe optimization. These constraints may partly explain the relatively weak cRNA or mRNA signals observed for certain genome segments. Experimentally derived structural information, such as SHAPE-MaP data, may help further optimize probe design (37).

Applying TriLock-FISH to infected lung tissue extends viral RNA imaging from cultured cells to native infection contexts, enabling direct investigation of viral RNA spatiotemporal dynamics in intact host tissues. In addition, the triple-lock probe design may be adaptable to other segmented negative-sense RNA viruses, such as Rift Valley fever virus and lymphocytic choriomeningitis virus (LCMV), although virus-specific probe design and experimental validation are required (38). In particular, the ambisense coding strategy of LCMV, in which viral mRNAs are transcribed from both the genomic and antigenomic RNAs, presents additional challenges for distinguishing RNA species. Although smFISH has been used to distinguish LCMV RNA species, the potential advantages of TriLock-FISH, including its use of only three probes per target to distinguish closely related RNA species, remain to be established in other viral systems (38).

In summary, TriLock-FISH provides a unique combination of specificity, genome-wide coverage, and spatial resolution for single-cell analysis of influenza virus RNA dynamics *in vitro* and *in vivo*. In particular, the ability to distinguish cRNA from mRNA *in situ* enables more precise spatial dissection of viral replication and transcription. It also enables direct comparison of viral RNA dynamics between cultured cells and native tissues, expanding opportunities for spatially resolved studies of RNA virus biology.

## MATERIALS AND METHODS

### Cells and viruses

The following IAV strains were used: A/Puerto Rico/8/1934 (H1N1) (PR8). The representative strain of Eurasian avian-like H1N1 swine influenza virus (EA2, GISAID accession number: EPI_ISL_539853, https://gisaid.org/) and the isogenic mutant EA2 R351K were also employed (39). Viruses were propagated in MDCK cells (ATCC) cultured in infection medium (MEM supplemented with 0.3% bovine serum albumin [BSA; Sigma-Aldrich], 1% P/S, 1% vitamins, 25 mM HEPES, and 1 µg/mL TPCK-treated trypsin [Sigma-Aldrich, 4352157]) at a multiplicity of infection (MOI) of 0.005. Viral stocks were passaged twice, sequence-verified by Sanger sequencing or next-generation sequencing, titrated by plaque assay on MDCK cells (pfu/mL), and stored at −80°C. All work with infectious viruses was conducted in approved Biosafety Level 2 laboratories.

### TriLock-FISH probe design and synthesis

The TriLock-FISH probe system for each target RNA consists of three oligonucleotides (P1–P3). P1 contains a 5′ region (20 nt) complementary to the class-defining region of the RNA (e.g., internal vRNA sequence, conserved 3′ end of cRNA or mRNA poly(A) junction), a central region (16 nt) complementary to P2, and a 3′ region (8 nt) complementary to P3. P2 comprises a 5′ region (16 nt) complementary to P1 and a 3′ region (8 nt) complementary to a segment-specific internal RNA sequence adjacent to the P1 binding site. P3 contains a 5′ region (16 nt) complementary to the segment-specific internal sequence immediately downstream of the P2 binding site, two tandem repeats (each 16 nt) serving as fluorescent detection probe binding sites separated by single adenine spacers for flexibility, and a 3′ region (8 nt) complementary to P1. All probe sequences targeting the eight segments of the PR8 strain for vRNA, cRNA, and mRNA are listed in Table S1. The probes designed to distinguish single-nucleotide differences are listed in Table S2. Probes for detection of *GAPDH* mRNA are listed in Table S3. Probes were synthesized commercially, and P2 and P3 were 5′-phosphorylated using T4 Polynucleotide Kinase (Vazyme, N102-01) to permit subsequent ligation.

### TriLock-FISH protocol

The TriLock-FISH assay was performed on fixed cells or tissue sections as described below, with minor optimizations for tissue samples. The core protocol consists of six sequential steps:

1. First hybridization: samples were incubated in hybridization buffer A (2× SSC, 10% formamide, 2× Denhardt’s solution [Aladdin, AA0230], 20 mM ribonucleoside vanadyl complexes [RVCs; BBI, B644221], 0.1 mg/mL salmon sperm DNA [Invitrogen, AM9680], and 0.1% Tween-20) containing a pre-annealed duplex of P1 and P2 probes (400 nM each) for 8 h at 37°C.
2. Wash: samples were washed twice with pre-warmed DEPC-PBSTR (DEPC-treated PBS with 0.05% Tween-20 and 0.1 U/μL RNase inhibitor) and once with 4× SSC in DEPC-PBSTR, each for 10 min at 37°C.
3. Second hybridization: samples were incubated in hybridization buffer B (2× SSC, 5% formamide, 2× Denhardt’s solution, 20 mM RVC, 0.1 mg/mL salmon sperm DNA, and 0.1% Tween-20) containing the P3 probe (800 nM) overnight at 37°C, followed by the same wash procedure as in step 2.
4. Ligation: samples were incubated in ligation buffer (1× SplintR ligase buffer, 1 U/μL SplintR ligase [NEB, M0375], 1.6 U/μL T4 DNA ligase [NEB, M0202], 0.1 mM ATP [NEB, P0756], 0.2 μg/μL BSA, and 0.1 U/μL RNase inhibitor) for 2 h at 16°C and then 2 h at 25°C, followed by three 5-min washes with DEPC-PBSTR.
5. Rolling circle amplification (RCA): samples were incubated in RCA buffer (1× Phi29 DNA polymerase buffer, 1 U/μL Phi29 DNA polymerase [Vazyme, N106-01], 1 mM dNTPs, 0.2 μg/μL BSA, and 5% glycerol) for 6–8 h at 30°C, followed by three 5-min washes with DEPC-PBST.
6. Fluorescent detection and imaging: Samples were incubated in detection buffer (2× SSC, 100 nM of each Alexa Fluor-labeled detection probe specific for the RCA product) for 2 h at 37°C. After washing, nuclei were counterstained with DAPI (0.5 μg/mL) and mounted. Images were acquired using a Nikon AX R NSPARC confocal microscope with a 60× oil immersion objective.

### Padlock probing protocol

Conventional cDNA padlock probe hybridization and direct RNA padlock probe hybridization were performed as described previously (30). For each method, a single padlock probe set targeting the NP-segment vRNA was used. In the cDNA padlock method, viral RNA was first reverse-transcribed into cDNA, which was then hybridized with the padlock probe. In the direct RNA padlock method, the padlock probe was hybridized directly to the target RNA, omitting the reverse transcription step. All padlock probe sequences are listed in Table S4.

### Specificity and validation assays

#### *In vitro *transcript transfection

To obtain defined PR8 NP vRNA, cRNA, and mRNA, we performed T7 *in vitro* transcription. Briefly, DNA templates were generated by PCR from the pHW2000 plasmid using forward primers carrying a T7 promoter. To mimic the poly(A) tail, the reverse primer for the mRNA template contained a stretch of 30 adenines. The three NP RNA species were synthesized using the TranscriptAid T7 High Yield Transcription Kit (Thermo Fisher, K0441). Then, MDCK cells were transfected with 50 ng of *in vitro*-transcribed, purified vRNA, cRNA, or mRNA (or a mixture thereof) using Lipomaster 3000 (Vazyme, TL301). The cells were fixed 4 h post-transfection for TriLock-FISH.

#### Pharmacological inhibition

MDCK cells were pretreated for 1 h with actinomycin D (10 µg/mL), cycloheximide (100 µg/mL), or DMSO (vehicle control). The cells were then infected with PR8 (MOI = 5) in the continued presence of the corresponding inhibitor or DMSO and fixed at 5 h post-infection (hpi) for analysis.

#### Drug treatment, replication, and cRNP stabilization assays

Drug treatment, replication, and cRNP stabilization assays were performed as previously described with minor modifications (24). For replication and cRNP stabilization assays, MDCK cells were transfected with pCAGGS plasmids expressing PR8 polymerase components (PB2:PB1:PA:NP = 2:2:1:4, where 1 = 80 ng) using Lipofectamine 3000 (Invitrogen, L3000015). WT PB1 was used for replication assays, PB1 D446Y for cRNP stabilization assays, and PB1 omission served as a negative control. At 24 h post-transfection, the cells were treated with actinomycin D (10 μg/mL) for 1 h, infected with PR8 (MOI = 5) in the presence of the drug, and fixed at 5 h post-infection (hpi) for analysis. For drug treatment alone, non-transfected cells were pretreated with actinomycin D (10 μg/mL), cycloheximide (100 μg/mL), or DMSO for 1 h before infection under the same conditions.

#### Single-nucleotide specificity test

MDCK cells were infected with either EA2 virus or the isogenic EA2 R351K mutant (MOI = 0.5) and fixed at 9 hpi for TriLock-FISH using probes designed to distinguish the single-nucleotide difference.

### Infection time-course and single-cell spatial RNA analysis

For genome-wide profiling, MDCK cells were infected with PR8 (MOI = 0.5) and fixed at 6 hpi. TriLock-FISH targeting all eight viral segments was conducted to generate single-cell spatial RNA profiles.

For spatiotemporal analysis, MDCK cells were infected with PR8 (MOI = 1) and fixed at 1, 2, 3, 4, 5, 6, 7, and 8 hpi. TriLock-FISH was performed to detect NP-segment vRNA, cRNA, and mRNA for single-cell quantification of RNA abundance and nuclear–cytoplasmic distribution.

### Mini-genome assay

HEK-293T cells (ATCC, cultured in Opti-MEM reduced serum medium supplemented with 5% FBS and 1% P/S) in confocal dishes were transfected using Lipofectamine 3000 with pHW2000 plasmids mixtures encoding PR8 polymerase components in the ratios 2:2:1:4, PB2:PB1:PA:NP, where 1 = 80 ng. The cells were fixed 24 h post-transfection and processed for TriLock-FISH detection of NP-derived RNAs.

### TriLock-FISH coupled with immunofluorescence

For combined detection, TriLock-FISH was performed first as described above. Following the final wash, samples were blocked with 5% BSA in PBS for 1 h and then incubated with a rabbit anti-influenza A virus NP primary antibody (GeneTex, GTX125989; 1:1,000) overnight at 37°C. After washing, samples were incubated with DyLight 405-conjugated goat anti-rabbit IgG secondary antibody (Abbkine, A23120; 1:1,000) for 1 h at 37°C, and imaged on a Nikon AX R NSPARC confocal microscope with 60× oil immersion objective.

### *In vivo* mouse infection and tissue processing

Female BALB/c mice (6–8 weeks old) were anesthetized and intranasally inoculated with 10^5^ PFU of PR8 virus in 20 µL PBS. At designated time points (8, 16, 24, and 72 hpi), mice were euthanized, and trachea, lungs, brain, and nasal turbinate were harvested. Portions of each tissue were homogenized for RNA extraction, and the remaining tissue was fixed in 4% PFA for 24 h, cryoprotected in 30% sucrose, embedded in optimal cutting temperature compound (OCT; SAKURA, 4583) at −80℃, and sectioned at 9 µm. Sections were post-fixed and processed for TriLock-FISH as described, with adaptations including a methanol dehydration step (−40°C for 15 min) and permeabilization with 2 mg/mL pepsin (37°C for 15 min).

### Quantitative RT-PCR (qPCR)

#### Strand-specific RT-qPCR for quantification of vRNA, cRNA, and mRNA

MDCK cells were cultured in confocal dishes, with each condition in triplicate. After infection, total RNA was extracted using the RNeasy Plus Mini Kit (QIAGEN, 74134) according to the manufacturer’s instructions. Quantification of vRNA, cRNA, and mRNA of eight segments was based on the tagged primer approach developed by Kawakami et al. (9). RT and qPCR primer sequences are listed in Table S5. For each sample, three separate reverse transcription reactions were performed using 200 ng total RNA and GoScript Reverse Transcriptase (Promega, A5003), each with a tagged primer specific for vRNA, cRNA, or viral mRNA. Tagged cDNA was quantified by real-time qPCR using PerfectStart green qPCR SuperMix (Transgen, AQ601). qPCR was performed at 94°C for 2 min, followed by 40 cycles of 94°C for 15 s and 60°C for 30 s. Absolute copy numbers were determined using standard curves generated from 10-fold serial dilutions (10^9^–10^3^ copies/μL) of synthetic viral RNA standards.

#### TaqMan qRT-PCR for viral M gene quantification in mouse tissues

Total RNA was extracted from homogenized mouse tissues (trachea, lung, nasal turbinate, and brain) using TRIzol. One microgram of total RNA was subjected to TaqMan qRT-PCR using TransScript Probe One-Step qRT-PCR SuperMix (Transgen, AQ221). The assay used a TaqMan probe (5′-6-FAM-TGCAGTCCTCGCTCACTGGGCACG-BHQ1-3′) and M gene-specific primers (forward, 5′-GACCRATCCTGTCACCTCTGAC-3′; reverse, 5′-AGGGCATTYTGGACAAAKCGTCTA-3′)(40). Absolute copy numbers of total M RNA were determined using standard curves generated from 10-fold serial dilutions of *in vitro*-transcribed M gene RNA standards.

### Immunohistochemistry (IHC)

Formalin-fixed, paraffin-embedded (FFPE) lung tissue was sectioned at 4 μm, deparaffinized in xylene, and rehydrated through graded ethanol to water. Antigen retrieval was performed in EDTA buffer (1 mM, pH 8.0) using heat-induced epitope retrieval. Endogenous peroxidase activity was quenched with 3% H_2_O_2_, and sections were blocked with 3% BSA for 30 min at room temperature. Slides were incubated with the anti-NP primary antibody (GeneTex, GTX125989) for 1 h at 37 ℃, followed by HRP-conjugated secondary antibodies for 1 h at room temperature. Signal was developed with DAB substrate, and sections were counterstained with hematoxylin.

### Histopathological examination

For histopathological examinations, the formalin-fixed lung tissues were dehydrated, embedded in paraffin, sectioned at 4 μm, and stained with hematoxylin and eosin (H&E) staining.

### Quantification and statistical analysis

Confocal image stacks were processed and analyzed using a custom pipeline based on the U-FISH deep learning model for signal dot decoding, cell segmentation, and counting (41). Raw fluorescence images were loaded using scikit-image and preprocessed with exposure normalization and Gaussian filtering to reduce noise. Transcript spots were detected using the U-FISH model with pretrained weights to generate per-pixel predictions and spot coordinates. The cells were segmented with Cellpose (default parameters), instance masks were labeled with skimage.measure.label, and cell centroids were obtained from region props. To assign transcripts to individual cells, a KDTree (scikit-learn) was constructed from cell centroids, and each detected spot was mapped to the nearest cell, with unmatched spots recorded as missing values. Custom scripts were used to count gene occurrences per cell and generate a cell-by-gene expression matrix. Visualization combined the raw image, Cellpose mask, spot coordinates, and spot–cell linkage to produce composite figures for quality control and spatial transcriptomics analyses.

For single-cell analysis, the parameters were quantified per cell: (i) counts of each RNA species in the nucleus and cytoplasm; (ii) nuclear and cytoplasmic distribution (N/C ratio); and (iii) the proportion of each RNA relative to total viral RNA. Cell-to-cell heterogeneity was assessed by calculating the Gini coefficient for RNA abundance and spatial distribution metrics across the cell population at each time point. For tissue sections, due to dense cellularity, a semi-quantitative analysis was performed by enumerating total RNA puncta across multiple random high-power fields (60×) per sample.

All statistical analyses were performed using GraphPad Prism 9. Data are presented as mean ± standard deviation (SD). For comparisons of vRNA, cRNA, and mRNA in one group, the paired Friedman test followed by Dunn’s *post hoc* test was used. To analyze the specificity of the probe in detecting point mutation, the unpaired Mann-Whitney test was used. For comparisons among multiple groups, the Kruskal–Wallis test followed by Dunn’s *post hoc* test was used. Statistical significance was determined by *P* values (<0.05 as shown). No data points were excluded from the analyses.

Single-cell heterogeneity of viral RNA abundance and subcellular distribution was quantified using the Gini coefficient (30, 42). For each condition/time point, per-cell measurements (RNA spot counts per cell, nucleus or cytoplasmic fraction which was defined as nuclear or cytoplasmic spots/total spots) were collected across all analyzed cells (*n*), sorted in ascending order (𝑥_1_ ≤… ≤ 𝑥*_n_*), and the Gini coefficient was computed as


$$G=\frac{\sum_{i=1}^{n}(2i-n-1)x_{i}}{n\sum_{i=1}^{n}x_{i}}$$

For conducting correlation analysis, the data were first tested for normality using GraphPad’s built-in normality test. Since the data did not follow a normal distribution, Spearman correlation analysis was employed.

Linear regression analyses were performed in GraphPad Prism using single-cell RNA spot counts. Simple linear regression was applied to each pairwise comparison, and the corresponding *R*^2^ values were calculated.

## Data Availability

The original images, quantitative data, and custom Python analysis scripts for viral RNA spot counts quantification and subcellular localization analysis are available on Zenodo (https://doi.org/10.5281/zenodo.22894252).

## SUPPLEMENTAL MATERIAL

Supplemental material mbio.01572-26-s0001.docx Supplemental figures and tables.

ASM does not own the copyrights to Supplemental Material that may be linked to, or accessed through, an article. The authors have granted ASM a non-exclusive, world-wide license to publish the Supplemental Material files. Please contact the corresponding author directly for reuse.
