# Supplementary material for: TriLock-FISH enables in situ discrimination of influenza cRNA and mRNA for single-cell analysis of viral replication and transcription
Source: mBio. 2026 Oct 22;17(11):e01572-26. doi: 10.1128/mbio.01572-26 (PMC13753831; doi:10.1128/mbio.01572-26)
Supplement: Supplementary file 1 [file mbio.01572-26-s0001.docx]

**Supplementary Data**

**TriLock-FISH enables in situ discrimination of influenza cRNA and mRNA for single-cell analysis of viral replication and transcription**

Lingkai Zhang^1,2,3^, Wei Chen^1,2,3^, Xinyu Gao^1,2,3^, Huaiyuan Cai^1,2,3^, Xu Zhou^1,2,3^, Rizwan Ullah^1,2,3^, Xinyi Pei^1,2,3^, Jinxia Dai^1^, Gang Cao^1,2,3,4^, Guiqing Peng^1,2,3,4^, Hongbo Zhou^1,2,3,4^, Wen Su^1,2,3,5*^

^1^National Key Laboratory of Agricultural Microbiology, College of Veterinary Medicine, Huazhong Agricultural University, Wuhan, Hubei, China.

^2^ Hubei Hongshan Laboratory, Wuhan, 430070, Hubei, China.

^3^ Frontiers Science Center for Animal Breeding and Sustainable Production, Wuhan, Hubei, China.

^4^ The Cooperative Innovation Center for Sustainable Pig Production, Huazhong Agricultural University, Wuhan, Hubei, China.

^5^ Shennongjia Science and Technology Innovation Center, Shennongjia Multidisciplinary Observation and Research Station of the Ministry of Agriculture and Rural Affairs, Huazhong Agricultural University, Wuhan, Hubei, China.

*Address correspondence to Wen Su, vetsu@mail.hzau.edu.cn.

Lingkai Zhang and Wei Chen contributed equally to this article. Author order was determined by seniority.

**Fig S1 Conventional padlock probing fails to unambiguously separate cRNA and mRNA, whereas TriLock-FISH enables specific three-RNA detection.** (A) Classical padlock probing with a single-round hybridization. Because cRNA and mRNA share near-identical internal sequences, padlock probes usually targeting internal regions report combined cRNA and mRNA signals rather than resolving the two species. Representative images are shown (vRNA: Alex-488, green; cRNA and mRNA: Alex-546, red; nuclei: DAPI, blue). Scale bars, 25 μm. (B) Two-round hybridization padlock probing using an optimized RCA primer to improve target engagement prior to padlock binding. The optimized RCA primer has a 20-nt sequence targeting the conserved cRNA 3′ terminus, or the mRNA poly(A) junction. (vRNA: Alex-488, green; cRNA: Alex-546, red; mRNA: Alex-647, magenta; nuclei: DAPI, blue). Scale bars, 25 μm. (C) TriLock-FISH strategy. A split-probe design is assembled by two sequential hybridization steps, and only correctly assembled complexes are circularized by SplintR ligase together with T4 DNA ligase, enabling specific detection of vRNA, cRNA, and mRNA. Scale bars, 25 μm.

**Fig S2 Detailed architecture of TriLock-FISH probe sets for strand-specific detection of IAV vRNA, cRNA and mRNA.** Example probe designs for vRNA (A), cRNA (B) and mRNA (C), using the PR8 NP segment as example. For each RNA species, the target sequence and the corresponding oligonucleotides (P1, P2 and P3) are shown with color-coded alignment to illustrate probe–target complementarity and probe–probe hybridization regions. P1 provides RNA-class specificity by recognizing a class-defining feature: an internal vRNA site (A), the conserved cRNA 3' terminus (B), or the mRNA 3' poly(A) junction (C). P2 and P3 bind adjacent segment-specific internal sequences to confer segment identity and assemble a ligation-competent complex. The loop region of P3 contains tandem repeats of the fluorescent detection-probe binding sequence, enabling visualization after rolling-circle amplification.

**Fig S3 Mechanistic basis for specificity in TriLock-FISH: representative mis-hybridization scenarios fail to ligate and amplify.** Schematic analysis of representative off-target and mismatched probe–target combinations using the PR8 NP probe sets as an example. Correct detection requires sequential assembly of P1–P2 during the first hybridization and recruitment of P3 during the second hybridization to form a ligation-competent complex. Only fully assembled complexes are circularized to generate an RCA template. In contrast, mismatched combinations—including NP mRNA probes targeting cRNA or vice versa (2-4), and NP probe sets encountering RNAs from other viral segments or host transcripts (5,6)—fail to undergo complete ligation (no ligation or half-ligation) and therefore do not support rolling-circle amplification.

**Fig S4 Systematic negative controls for TriLock-FISH.** (A-C) Omission controls showing no detectable signal when P1, P2, or P3 is omitted from the TriLock-FISH reaction. (D-E) Mismatch controls showing no signal with a non-complementary P1 (D) or with mismatched P2 and P3 (E). (F) Off-target control using a non-target RNA sequence, showing no detectable amplification. (G) Positive control using the complete, correctly matched probe set, yielding robust signals. All assays were performed in MDCK cells targeting endogenous GAPDH mRNA. Nuclei are stained with DAPI (blue). Scale bars, 25 μm.

**Fig S5 Optimization of hybridization conditions to improve TriLock-FISH efficiency.** (A) Effect of Denhardt’s solution concentration (1 - 4×) on TriLock-FISH signal yield. Representative images (left) and per-cell spot counts (right) are shown. Error bars represent the mean ± s.d. Scale bars, 25 μm. (B) Effect of formamide concentration in the first and second hybridization buffers (1^st^ concentration – 2^nd^ concentration, indicated in each panel) on TriLock-FISH signal yield. Optimizations were performed using endogenous GAPDH mRNA as a stable cellular target. TriLock-FISH puncta are shown in magenta and nuclei are stained with DAPI (blue). Error bars represent the mean ± s.d. Scale bars, 25 μm.

**Fig S6 Comparison of TriLock-FISH with established padlock probe-based methods.** (A) Schematic illustration of conventional cDNA padlock probe hybridization, direct RNA padlock probe hybridization, and TriLock-FISH. Following hybridization to the target, the probes are ligated and subjected to rolling circle amplification (RCA), generating rolling circle products (RCPs) that are detected as fluorescent spots. (B) Representative images of IAV NP vRNA detected by the three methods in PR8-infected MDCK cells (MOI = 1, 8 hpi). Scale bars, 25 μm. (C) Quantification of vRNA spot counts per cell. P values were calculated using the Kruskal–Wallis test followed by Dunn’s multiple-comparisons test. Data are representative of two independent experiments.

**Fig S7 Validation of NP localization with and without TriLock-FISH.** Representative images of PR8-infected MDCK cells (MOI = 1) at 8 dpi analyzed by TriLock-FISH coupled with immunofluorescence (IFA; upper row) or IFA alone (lower row). Enlarged views of the boxed regions are shown. NP vRNA, Alexa 488 (green); NP cRNA, Alexa 546 (red); NP mRNA, Alexa 647 (magenta); nucleoprotein, DyLight 405 (yellow). Scale bars, 25 μm.

**Fig S8 Detection of an endogenous host transcript by TriLock-FISH.** Representative images showing detection of GAPDH mRNA in MDCK cells. GAPDH signals are shown in magenta; nuclei are stained with DAPI (blue). Scale bars, 25 μm.

**Fig S9 Additional quantification of NP-segment RNA dynamics and nuclear–cytoplasmic partitioning**. (A) Mean per-cell spot counts over time. Error bars represent the mean ± s.d. (B) Mean nuclear fraction (nuclear spot/total spot) over time. Error bars represent the mean ± s.d. (C) Violin plots showing the distribution of per-cell spot counts at each time point. (D) Violin plots showing the distribution of nuclear fractions at each time point. (E) Stacked bar plots summarizing the mean nuclear versus cytoplasmic contributions for each RNA species across the time course. (F) Pairwise relationships between cytoplasmic cRNA (ccRNA) abundance and vRNA, cRNA, or mRNA abundance at the single-cell level. Each dot represents one cell, and lines indicate linear fits.

**Fig S10 Single-cell 3D embeddings of infection states based on RNA distribution**. 3D scatter plot of the same cells embedded by subcellular distribution metrics. Left, all cells colored by time post-infection. Right, time-sliced views illustrating continuous changes in RNA localization over the course of infection.

**Fig S11 Time-resolved distributions of viral RNA abundance and subcellular localization at the single-cell level.** (A–C) Single-cell distributions of vRNA (A), cRNA (B), and mRNA (C), respectively, at the indicated times post infection (hpi). Each dot represents one cell. Data from the indicated time point are shown in color, while data from other time points are shown in gray.

**Fig S12 Time-resolved pairwise correlations among vRNA, cRNA, and mRNA abundances.**

(A-C) Pairwise relationships between vRNA and cRNA (A), vRNA and mRNA (B), and cRNA and mRNA (C) at each time point. Each dot represents one cell, and lines indicate linear fits with corresponding equations and R² values.

(D) Temporal trends of fitted slopes for each RNA pair, summarizing the relative changes in pairwise abundance relationships over time.

**Table S1 Probe sequences targeting eight segments of the PR8 strain for vRNA, cRNA, and mRNA.**

| RNA | Segment | Probe (5'-3') |
| --- | --- | --- |
| vRNA | **vP1** | |
|  | HA | CGGATGGCTCTTGGGAAACCAAGGACTAAGGTAAGCCCAATCGCA |
|  | M | ATAGCACAGAGACTTGAAGAAGTTCTTGCATGAGGAGCTACAGAC |
|  | NA | AGCCCTTTATTTCATGTTCACATTACAGTGGCTGGAAGGCATCT |
|  | NP | TCCAGGGGCGGGGAGTCTTCAGCAATGATAGGAAGAGCCGACATC |
|  | NS | GCGTTACCTAACTGACATGAAGATGTTACTCAGGTCCTTGTCTCG |
|  | PA | GCCTATGTGGATGGATTCGAAGCGACTGATAGTGAGATTGTGGAG |
|  | PB1 | AACACCGAAACTGGAGCACCAGCATGTTATGCGGAGGCAGTGTTG |
|  | PB2 | GCTGCAGTATCAGCAGATCCAAAGAGGTTAGCGTGGTGCACTTCT |
|  | **vP2** | |
|  | HA | GGCTTACCTTAGTCCTCAGAATGC |
|  | M | CTCCTCATGCAAGAACTGTCTTTG |
|  | NA | TCCAGCCACTGTAATGTCACTTGG |
|  | NP | CTCTTCCTATCATTGCGAGCTCTC |
|  | NS | GGACCTGAGTAACATCCTCTTGAG |
|  | PA | TCTCACTATCAGTCGCACCGAACG |
|  | PB1 | CCTCCGCATAACATGCGCAACTCA |
|  | PB2 | ACCACGCTAACCTCTTACTAGCAT |
|  | **vP3** | |
|  | HA | GACCCACTGCTTCCAGATAGTCGCAACTCTGAAATAGTCGCAACTCTGAAATGCGATTG |
|  | M | CAGGGAAGAACACCGAAGGCACTTGAGCTAGAAAGGCACTTGAGCTAGAAAGTCTGTAG |
|  | NA | AATGCAGGACCTTTTTAGTGCTTGAACTGGTCTAGTGCTTGAACTGGTCTAAGATGCCT |
|  | NP | GGACGAAAAGGCAGCGATATGTGACCGGGTACAATATGTGACCGGGTACAAGATGTCGG |
|  | NS | GAAATGTCAAGGGACTATATCTGACGCGAACTGATATCTGACGCGAACTGACGAGACAA |
|  | PA | GCTACATTGAGGGCAAAGTCGACCTACCCATAAAGTCGACCTACCCATAAACTCCACAA |
|  | PB1 | ACCCGATTGATGGGCCAGAGCGTTCGACTCCAAAGAGCGTTCGACTCCAAACAACACTG |
|  | PB2 | CTTTATTGGAGATGTGATCGATAGCGATCACCAATCGATAGCGATCACCAAAGAAGTGC |
|  | **vRNA fluorescent detection probe** | |
|  | HA | TAGTCGCAACTCTGAA |
|  | M | GGCACTTGAGCTAGAA |
|  | NA | GTGCTTGAACTGGTCT |
|  | NP | TATGTGACCGGGTACA |
|  | NS | TATCTGACGCGAACTG |
|  | PA | GTCGACCTACCCATAA |
|  | PB1 | GAGCGTTCGACTCCAA |
|  | PB2 | TCGATAGCGATCACCA |
| cRNA | **cP1** | |
|  | HA | AGTAGAAACAAGGGTGTTTTAGTGAGGACCTTACTCTGACCTAGT |
|  | M | AGTAGAAACAAGGTAGTTTTATGAGTGGCAAGATAGCATCGTTGC |
|  | NA | AGTAGAAACAAGGAGTTTTTAGCCAGTTTGAATGCCACCGATACT |
|  | NP | AGTAGAAACAAGGGTATTTTACGAGAGTTATCGTTGCCTCGACCA |
|  | NS | AGTAGAAACAAGGGTGTTTTATGCCTATGGAAGAGACTTGCATCG |
|  | PA | AGTAGAAACAAGGTACTTTTAATGGCGATATCTTACCAGCACATG |
|  | PB1 | AGTAGAAACAAGGCATTTTTACCTGACATGCTTATGGGAGCTCAT |
|  | PB2 | AGTAGAAACAAGGTCGTTTTAGCAGTATCATCTCGGACAGCACAT |
|  | **cP2** | |
|  | HA | AGAGTAAGGTCCTCACTCCTCATA |
|  | M | GCTATCTTGCCACTCATTACTCCA |
|  | NA | TGGCATTCAAACTGGCTGAACAGA |
|  | NP | GCAACGATAACTCTCGTCTTTAAT |
|  | NS | GTCTCTTCCATAGGCATTAGTACT |
|  | PA | GGTAAGATATCGCCATTTGGACAG |
|  | PB1 | CCATAAGCATGTCAGGTCATGAAG |
|  | PB2 | TCCGAGATGATACTGCTAAACTAT |
|  | **cP3** | |
|  | HA | TTTCTGAAATTCTAATACAGATAGGACTAGTCAACAGATAGGACTAGTCAAACTAGGTC |
|  | M | GCTCTATGCTGACAAAACCTCGATTAACATCGCACCTCGATTAACATCGCAGCAACGAT |
|  | NA | CTACTTGTCAATGGTTAGATCTCGAGAACGTCAAGATCTCGAGAACGTCAAAGTATCGG |
|  | NP | TGTCGTACTCCTCTGCAGTCTATTAGTGGAGCCAGTCTATTAGTGGAGCCATGGTCGAG |
|  | NS | AAATAAGCTGAAACGAAGCAATTAGCGATGGCAAGCAATTAGCGATGGCAACGATGCAA |
|  | PA | TATGGATAGCAAATAGAGTTGCAGTAAGTCGTGAGTTGCAGTAAGTCGTGACATGTGCT |
|  | PB1 | GACAAGCTAAATTCACAGACAGGTCTCTCGATAAGACAGGTCTCTCGATAAATGAGCTC |
|  | PB2 | TCGACACTAATTGATGAGTATCAGTGCGTACATAGTATCAGTGCGTACATAATGTGCTG |
|  | **cRNA fluorescent detection probe** | |
|  | HA | CAGATAGGACTAGTCA |
|  | M | CCTCGATTAACATCGC |
|  | NA | GATCTCGAGAACGTCA |
|  | NP | GTCTATTAGTGGAGCC |
|  | NS | GCAATTAGCGATGGCA |
|  | PA | GTTGCAGTAAGTCGTG |
|  | PB1 | GACAGGTCTCTCGATA |
|  | PB2 | GTATCAGTGCGTACAT |
| mRNA | **mP1** | |
|  | HA | TTTTTTTTTTTTTTTTTTCTAGCGGAACTATCTACACTGTGAGGA |
|  | M | TTTTTTTTTTTTTTTTTTACACTAGACAATCTGTGGCAGACCGAA |
|  | NA | TTTTTTTTTTTTTTTTTTGAAACAGGTTAGGTACTCCTCTGTACC |
|  | NP | TTTTTTTTTTTTTTTTTTCTACGTTCAAGGGAGACTTGGACTCGC |
|  | NS | TTTTTTTTTTTTTTTTTTAGATTTGTATGGCTGCTGGCAACGAAC |
|  | PA | TTTTTTTTTTTTTTTTTTGGAAGCACGTTCGCACAGACAGGTGAT |
|  | PB1 | TTTTTTTTTTTTTTTTTTCAAACGAGGTCGGAAATGGGTCCTTGA |
|  | PB2 | TTTTTTTTTTTTTTTTTTAAACTCGTCACCATACGTTGCGATGAT |
|  | **mP2** | |
|  | HA | GTGTAGATAGTTCCGCCATATTTC |
|  | M | GCCACAGATTGTCTAGTCCAGCTC |
|  | NA | GGAGTACCTAACCTGTACAGACTA |
|  | NP | AAGTCTCCCTTGAACGTTAATTGT |
|  | NS | CCAGCAGCCATACAAATACTAAAT |
|  | PA | TCTGTGCGAACGTGCTACAGTATG |
|  | PB1 | CCATTTCCGACCTCGTTGAAGGAC |
|  | PB2 | AACGTATGGTGACGAGACTATTCG |
|  | **mP3** | |
|  | HA | TGAAATTCTAATCTCAAATGAGATCGTCATCGTAATGAGATCGTCATCGTATCCTCACA |
|  | M | TATGCTGACAAAATGAAATGCTGCTGTAGTACTAATGCTGCTGTAGTACTATTCGGTCT |
|  | NA | CTTGTCAATGGTGAATACTGACTAACGACACCAACTGACTAACGACACCAAGGTACAGA |
|  | NP | CGTACTCCTCTGCATTATACCCGAGATAGAGTCATACCCGAGATAGAGTCAGCGAGTCC |
|  | NS | AAGCTGAAACGAGAAAAAGACGTAATGACAGCGAAGACGTAATGACAGCGAGTTCGTTG |
|  | PA | GATAGCAAATAGTAGCATATGCGAGGGCTTCAAATATGCGAGGGCTTCAAAATCACCTG |
|  | PB1 | AAGCTAAATTCACTATAAGTGTTAGCAGGTTGCAAGTGTTAGCAGGTTGCATCAAGGAC |
|  | PB2 | ACACTAATTGATGGCCAGGTTGTACGCATATGCAGGTTGTACGCATATGCAATCATCGC |
|  | **mRNA fluorescent detection probe** | |
|  | HA | ATGAGATCGTCATCGT |
|  | M | ATGCTGCTGTAGTACT |
|  | NA | CTGACTAACGACACCA |
|  | NP | TACCCGAGATAGAGTC |
|  | NS | AGACGTAATGACAGCG |
|  | PA | TATGCGAGGGCTTCAA |
|  | PB1 | AGTGTTAGCAGGTTGC |
|  | PB2 | GGTTGTACGCATATGC |

**Table S2 Probe sequences designed to distinguish single-nucleotide difference.**

| Target | Probe (5'-3') | |
| --- | --- | --- |
| EA2 | P1 | TCTGAGAGTGTCAAGTTTCAAGCAATGATAGGAAGAGCCGACATC |
|  | P2 | CTCTTCCTATCATTGCAGGGACAA |
|  | P3 | AAGTGGTCCCAAGAGGATATGTGACCGGGTACAATATGTGACCGGGTACAAGATGTCGG |
| EA2 R351K | P1 | GAGTGTCAAGTTTCATCAGAACGTTCAAGGGAGACTTGGACTCGC |
|  | P2 | AAGTCTCCCTTGAACGGGGACAAG |
|  | P3 | AGTGGTCCCAAGAGGAATACCCGAGATAGAGTCATACCCGAGATAGAGTCAGCGAGTCC |

**Table S3 Probes for detection of dog *GAPDH* mRNA**

| Target | Probe (5'-3') | |
| --- | --- | --- |
| Dog *GAPDH* mRNA | P1 | CCTCCACGATGCCGAAGTGGACGAGAGTTATCGTTGCCTCGACCA |
|  | P2 | GCAACGATAACTCTCGTCATGGAT |
|  | P3 | GACTTTGGCTAGAGGAATACCCGAGATAGAGTCATACCCGAGATAGAGTCATGGTCGAG |

**Table S4 Padlock probes for detection of NP vRNA**

| Target | Probe (5'-3') | | |
| --- | --- | --- | --- |
| NP cDNA Padlock | Padlock probe | GAGAGCTCGAAGATATGTGACCGGGTACAATATGTGACCGGGTACATGCCTTTTCGTCC |  |
|  | RCA Primer | CGATCGGGCTCGCGATGTACCCGGTCACATA |  |
| NP RNA Padlock | Padlock probe | GGACGAAAAGGCATATGTGACCGGGTACAATATGTGACCGGGTACATCTTCGAGCTCTC |  |
|  | RCA Primer | CAGGGGCGGGGAGATGTACCCGGTCACATA |  |

**Table S5 RT and qPCR primers used for strand-specific quantification of PR8**

| Target | | Purpose | Primer name | Probe (5'-3') |
| --- | --- | --- | --- | --- |
| PB2 | vRNA | Reverse transcription | vPB2_tagRT_P | GGCCGTCATGGTGGCGAACACAAAAACCACCGTGGACC |
|  |  | Real-time PCR | vRNA_Tag | GGCCGTCATGGTGGCGAA |
|  |  |  | vPB2_qPCR_R | TCTCAGGAATCATTTCCGTTATCC |
|  | cRNA | Reverse transcription | cPB2_tagRT_P | GCTAGCTTCAGCTAGGCATCAGTAGAAACAAGGTCGTTTTTAAAC |
|  |  | Real-time PCR | cRNA_Tag | GCTAGCTTCAGCTAGGCATC |
|  |  |  | cmPB2_qPCR_R | AGGCTAATGTGCTAATTGGG |
|  | mRNA | Reverse transcription | mPB2_tagRT_P | CCAGATCGTTCGAGTCGTTTTTTTTTTTTTTTTTAAACTATTCGA |
|  |  | Real-time PCR | mRNA_Tag | CCAGATCGTTCGAGTCGTTTTTT |
|  |  |  | cmPB2_qPCR_R | AGGCTAATGTGCTAATTGGG |
| PB1 | vRNA | Reverse transcription | vPB1_tagRT_P | GGCCGTCATGGTGGCGAAACTGTCAACAGGACACATCAG |
|  |  | Real-time PCR | vRNA_Tag | GGCCGTCATGGTGGCGAA |
|  |  |  | vPB1_qPCR_R | AAGCCATCGCCTCCAATAC |
|  | cRNA | Reverse transcription | cPB1_tagRT_P | GCTAGCTTCAGCTAGGCATCAGTAGAAACAAGGCATTTTTTCATG |
|  |  | Real-time PCR | cRNA_Tag | GCTAGCTTCAGCTAGGCATC |
|  |  |  | cmPB1_qPCR_R | GAGCCCGAATTGATGCACGGATTG |
|  | mRNA | Reverse transcription | mPB1_tagRT_P | CCAGATCGTTCGAGTCGTTTTTTTTTTTTTTTTTTTCATGAAGGA |
|  |  | Real-time PCR | mRNA_Tag | CCAGATCGTTCGAGTCGTTTTTT |
|  |  |  | cmPB1_qPCR_R | GAGCCCGAATTGATGCACGGATTG |
| PA | vRNA | Reverse transcription | vPA_tagRT_P | GGCCGTCATGGTGGCGAATTGTCGAGCTTGCGGAAA |
|  |  | Real-time PCR | vRNA_Tag | GGCCGTCATGGTGGCGAA |
|  |  |  | vPA_qPCR_R | TATTGACTCGCCTTGCTCATT |
|  | cRNA | Reverse transcription | cPA_tagRT_P | GCTAGCTTCAGCTAGGCATCAGTAGAAACAAGGTACTTTTTTGGA |
|  |  | Real-time PCR | cRNA_Tag | GCTAGCTTCAGCTAGGCATC |
|  |  |  | cmPA_qPCR_R | GCTATATGAAGCAATTGAGGAGTG |
|  | mRNA | Reverse transcription | mPA_tagRT_P | CCAGATCGTTCGAGTCGTTTTTTTTTTTTTTTTTGGACAGTATGG |
|  |  | Real-time PCR | mRNA_Tag | CCAGATCGTTCGAGTCGTTTTTT |
|  |  |  | cmPA_qPCR_R | GCTATATGAAGCAATTGAGGAGTG |
| HA | vRNA | Reverse transcription | vHA_tagRT_P | GGCCGTCATGGTGGCGAAACAGCCACAACGGAAAACTATG |
|  |  | Real-time PCR | vRNA_Tag | GGCCGTCATGGTGGCGAA |
|  |  |  | vHA_qPCR_R | TCTCAGAGTTTGGTGTTTCT |
|  | cRNA | Reverse transcription | cHA_tagRT_P | GCTAGCTTCAGCTAGGCATCAGTAGAAACAAGGGTGTTTTTCCTC |
|  |  | Real-time PCR | cRNA_Tag | GCTAGCTTCAGCTAGGCATC |
|  |  |  | cmHA_qPCR_R | AGATTCTGGCGATCTACTCAACTG |
|  | mRNA | Reverse transcription | mHA_tagRT_P | CCAGATCGTTCGAGTCGTTTTTTTTTTTTTTTTTTTCCTCATATC |
|  |  | Real-time PCR | mRNA_Tag | CCAGATCGTTCGAGTCGTTTTTT |
|  |  |  | cmHA_qPCR_R | AGATTCTGGCGATCTACTCAACTG |
| NP | vRNA | Reverse transcription | vNP_tagRT_P | GGCCGTCATGGTGGCGAAGAGAATGGACGAAAAACAAGAATTGC |
|  |  | Real-time PCR | vRNA_Tag | GGCCGTCATGGTGGCGAA |
|  |  |  | vNP_qPCR_R | CTCAATATGAGTGCAGACCGTGCT |
|  | cRNA | Reverse transcription | cNP_tagRT_P | GCTAGCTTCAGCTAGGCATCAGTAGAAACAAGGGTATTTTTCTTT |
|  |  | Real-time PCR | cRNA_Tag | GCTAGCTTCAGCTAGGCATC |
|  |  |  | cmNP_qPCR_R | ACCAGAAGATGTGTCTTTCCAG |
|  | mRNA | Reverse transcription | mNP_tagRT_P | CCAGATCGTTCGAGTCGTTTTTTTTTTTTTTTTTCTTTAATTGTC |
|  |  | Real-time PCR | mRNA_Tag | CCAGATCGTTCGAGTCGTTTTTT |
|  |  |  | cmNP_qPCR_R | ACCAGAAGATGTGTCTTTCCAG |
| NA | vRNA | Reverse transcription | vNA_tagRT_P | GGCCGTCATGGTGGCGAATACTATAATGACTGATGGCCCGAGT |
|  |  | Real-time PCR | vRNA_Tag | GGCCGTCATGGTGGCGAA |
|  |  |  | vNA_qPCR_R | ACATCACTTTGCCGGTATCAGGGT |
|  | cRNA | Reverse transcription | cNA_tagRT_P | GCTAGCTTCAGCTAGGCATCAGTAGAAACAAGGAGTTTTTTGAAC |
|  |  | Real-time PCR | cRNA_Tag | GCTAGCTTCAGCTAGGCATC |
|  |  |  | cmNA_qPCR_R | AACAATCTGGACTAGTGCGAGCAG |
|  | mRNA | Reverse transcription | mNA_tagRT_P | CCAGATCGTTCGAGTCGTTTTTTTTTTTTTTTTTGAACAGACTAC |
|  |  | Real-time PCR | mRNA_Tag | CCAGATCGTTCGAGTCGTTTTTT |
|  |  |  | cmNA_qPCR_R | AACAATCTGGACTAGTGCGAGCAG |
| M | vRNA | Reverse transcription | vM_tagRT_P | GGCCGTCATGGTGGCGAAAGCCGAGATCGCACAGAGACTT |
|  |  | Real-time PCR | vRNA_Tag | GGCCGTCATGGTGGCGAA |
|  |  |  | vM_qPCR_R | GAGCGTGAACACAAATCCTA |
|  | cRNA | Reverse transcription | cM_tagRT_P | GCTAGCTTCAGCTAGGCATCAGTAGAAACAAGGTAGTTTTTTACT |
|  |  | Real-time PCR | cRNA_Tag | GCTAGCTTCAGCTAGGCATC |
|  |  |  | cmM_qPCR_R | ACTGAAAGGAGGGCCTTCTACGGA |
|  | mRNA | Reverse transcription | mM_tagRT_P | CCAGATCGTTCGAGTCGTTTTTTTTTTTTTTTTTTTACTCCAGCT |
|  |  | Real-time PCR | mRNA_Tag | CCAGATCGTTCGAGTCGTTTTTT |
|  |  |  | cmM_qPCR_R | ACTGAAAGGAGGGCCTTCTACGGA |
| NS | vRNA | Reverse transcription | vNS_tagRT_P | GGCCGTCATGGTGGCGAACGAGTTGCAGACCAAGAACTA |
|  |  | Real-time PCR | vRNA_Tag | GGCCGTCATGGTGGCGAA |
|  |  |  | vNS_qPCR_R | TTTCAGAATCCGCTCCACTATC |
|  | cRNA | Reverse transcription | cNS_tagRT_P | GCTAGCTTCAGCTAGGCATCAGTAGAAACAAGGGTGTTTTTTATT |
|  |  | Real-time PCR | cRNA_Tag | GCTAGCTTCAGCTAGGCATC |
|  |  |  | cmNS_qPCR_R | GAAGAAGTGAGACACAAACTGAAG |
|  | mRNA | Reverse transcription | mNS_tagRT_P | CCAGATCGTTCGAGTCGTTTTTTTTTTTTTTTTTTTATTACTAAA |
|  |  | Real-time PCR | mRNA_Tag | CCAGATCGTTCGAGTCGTTTTTT |
|  |  |  | cmNS_qPCR_R | GAAGAAGTGAGACACAAACTGAAG |
